# Genomic Characterization of *Salmonella* Minnesota Clonal Lineages Associated with Poultry Production in Brazil

**DOI:** 10.3390/ani10112043

**Published:** 2020-11-05

**Authors:** Diéssy Kipper, Laura M. Carroll, Andrea K. Mascitti, André F. Streck, André S. K. Fonseca, Nilo Ikuta, Vagner R. Lunge

**Affiliations:** 1 Laboratório de Diagnóstico Molecular, Universidade Luterana do Brasil (ULBRA), Canoas, Rio Grande do Sul 92425-020, Brazil; diessykipper@hotmail.com (D.K.); andreakaroline88@hotmail.com (A.K.M.); 2 Department of Food Science, Cornell University, Ithaca, New York, NY 14850, USA; laura.carroll@embl.de; 3 Laboratório de Diagnóstico em Medicina Veterinária, Universidade de Caxias do Sul (UCS), Caxias do Sul, Rio Grande do Sul 95070-560, Brazil; afstreck@ucs.br; 4 Simbios Biotecnologia, Cachoeirinha, Rio Grande do Sul 94940-030, Brazil; fonseca@simbios.com.br (A.S.K.F.); ikuta@simbios.com.br (N.I.)

**Keywords:** phylodynamic, whole-genome sequencing (WGS), chicken, antimicrobial resistance genes, virulence genetic cluster

## Abstract

**Simple Summary:**

*Salmonella* is a leading cause of foodborne illnesses and a global public health concern. *Salmonella enterica* serotype Minnesota has been increasingly isolated from Brazilian poultry farms. The present study investigated the phylogenetic relationships, evolution and genetic characteristics of *S.* Minnesota isolates from Brazilian poultry farms. The results demonstrated two main *S.* Minnesota lineages in the poultry production chain from Brazil, both presenting genes for antibiotic resistance and virulence. The present study also provides insights into the temporal evolution, population structure, and genetic characteristics of the two *S.* Minnesota lineages disseminated in Brazilian poultry farms.

**Abstract:**

*Salmonella* serotype Minnesota has been increasingly detected in Brazilian poultry farms and food products (chicken meat, eggs) in recent years. In addition, *S.* Minnesota isolates from poultry are generally resistant to several antibiotics and persistent in farm environments. The present study aimed to assess phylogenomic diversity of *S*. Minnesota isolates from the poultry production chain in Brazil. In total, 107 worldwide *S*. Minnesota whole genomes (including 12 from Brazil) were analyzed using a comparative approach. Phylogenetic analysis demonstrated two clades more related to poultry production in Brazil: *S*. Minnesota poultry lineages I and II (SM-PLI and SM-PLII). Phylodynamic analysis demonstrated that SM-PLI had a common ancestor in 1915, while SM–PLII originated circa 1971. SM-PLII encompassed a higher number of isolates and presented a recent increase in effective population size (mainly from 2009 to 2012). Plasmids IncA/C2 and ColRNA, antimicrobial resistance genes (*aph(3′)-Ia*, *blaCMY-2*, *qnrB19*, *sul2*, and *tet(A)*) and mainly a virulence genetic cluster (including the yersiniabactin operon) were detected in isolates from SM-PLI and/or SM-PLII. This study demonstrates the dissemination of two distinct *S*. Minnesota lineages with high resistance to antibiotics and important virulence genetic clusters in Brazilian poultry farms.

## 1. Introduction

*Salmonella* is a leading cause of foodborne illnesses and a global public health concern [1]. It is a Gram-negative, rod-shaped facultative anaerobic bacterium belonging to the family Enterobacteriaceae. The genus *Salmonella* is divided into two species (*Salmonella enterica* and *Salmonella bongori*), but it is also classified into several serotypes by immunological assays. So far, more than 2600 serotypes have already been identified within the *Salmonella* genus worldwide, many of them associated with enteric and systemic diseases in domestic animals and humans [2].

In poultry production, *Salmonella* is disseminated into the flocks and farm environments via the avian feces. Other animals may become infected through contaminated poultry litter or close contact with an infected bird. High *Salmonella* bacterial content in the enteric tract of broilers and layers can result in the contamination of chicken meat, eggs, and other poultry products in slaughterhouses [3].

*Salmonella enterica* serotype Minnesota was first isolated in a turkey farm from Minnesota (United States) in 1936 [4]. Since then, this serotype has been detected in different sources, including the natural environment, plants, animal-producing farms, and foods [5,6,7]. Poultry products seem to be an important source of human infection with this bacterial foodborne pathogen. In addition, bacteriological analyses have demonstrated that *S.* Minnesota isolates are resistant to antibiotics, including extended-spectrum cephalosporins [8]. 

In Brazil, *S.* Minnesota has been frequently detected on poultry farms (10% to 30%) since the beginning of this century [9,10]. In addition to the farm environment, *S.* Minnesota has also been detected in slaughterhouses and foods [10]. A very high frequency (reaching 86.6%) was observed in several poultry farms from the Brazilian Center West Region around 2010 [6]. *S*. Minnesota was even detected in chicken carcasses in Brazilian markets, as well as in poultry products exported to Portugal [8,11]. The present study aimed to evaluate the population structure, phylogenetic relationships, temporal evolution, and genetic characteristics (e.g., plasmids and antibiotic resistance and virulence genes) of the *S.* Minnesota strains isolated from Brazilian poultry production chain.

## 2. Material and Methods 

### 2.1. Bacterial Isolates and Molecular Biology Assays

Three *S.* Minnesota isolates were obtained from poultry farms in the state of Mato Grosso do Sul, Center West Region from Brazil, in 2018 (Table S1). Single colonies of each isolate were removed from xylose lysine desoxycholate (XLD) agar plates and placed in brain heart infusion (BHI) broth, following overnight incubation at 35 °C. DNA was extracted using a commercial method according to the manufacturer’s instructions (NewGene, Simbios Biotecnologia, Cachoeirinha, RS, Brazil) and was further characterized by PCR/sequencing to confirm the genus *Salmonella* and serotype Minnesota with the following experimental approaches: (i) *invA* real-time PCR for *Salmonella* detection (reagents NewGene SALAmp, Simbios Biotecnologia, Cachoeirinha, RS, Brazil), and (ii) *rrn*H operon intergenic sequence ribotyping (ISR) analysis for serotype assignment [12].

### 2.2. Whole-Genome Sequencing

The PureLink^®^ Genomic DNA Mini Kit was used to extract DNA following the manufacturer’s instructions (Thermo Fisher Scientific, Waltham, MA, USA), and DNA was visualized on a 2% agarose gel stained with ethidium bromide. Sequencing libraries were prepared using the Nextera XT kit (Illumina, Inc., San Diego, CA, USA). DNA concentration was adjusted to 0.2 ng/µL, and sequencing was performed on an Illumina NextSeq platform, using 150 bp paired-end reads (Wadsworth Center, New York State Department of Health (NYSDOH), Albany, NY, USA). 

### 2.3. Acquisition of Genomic Data and in Silico Identification of Genetic Elements

Trimmomatic version 0.33 [13] was used to trim raw Illumina sequence reads and remove low-quality bases. The quality of the resulting trimmed reads was assessed using FastQC version 0.11.2 [14] prior to de novo assembly using SPAdes version 3.6.0 [15]. The quality of draft genomes was evaluated using QUAST version 5.0.2 [16], and per-base average coverage was estimated by mapping each isolate’s trimmed reads to its respective assembled contigs using BBmap version 38.26 [17] and Samtools version 1.9 [18]. Assemblies were annotated using Prokka version 1.12 [19]. SISTR version 0.3.1 [20] was used to perform in silico serotyping, and each isolate was also assigned to a sequence type (ST) using seven-gene multi-locus sequence typing (MLST; https://github.com/tseemann/mlst).

The isolates sequenced here were analyzed together with publicly available *S.* Minnesota genomes. Among the genome sequences available in the National Center for Biotechnology Information’s (NCBI’s) Sequence Read Archive (SRA) database [21], 104 were selected to represent as many countries, sources, and years as possible and were downloaded. These whole-genome sequences (WGS), plus the three new Brazilian *S.* Minnesota sequenced here, were used in all analyses (Table S1). 

ABRicate version 0.8 (https://github.com/tseemann/abricate) was used to detect antimicrobial resistance genes, virulence factors, and plasmid replicons in each assembled genome, using the ResFinder database [22], Virulence Factor Database (VFDB) [23], and PlasmidFinder [24] database, respectively (accessed 11 June 2018). *Salmonella* pathogenicity islands (SPIs) were additionally detected in each genome using the nucleotide Basic Local Alignment Search Tool (BLASTN) [25]. For all searches, minimum nucleotide identity and coverage thresholds of 75% and 50% were used, respectively. 

### 2.4. Identification of Single-Nucleotide Polymorphisms (SNPs) and Construction of Maximum Likelihood Phylogenies

Single-nucleotide polymorphisms (SNPs) were identified among all 107 *S.* Minnesota assemblies (i.e., the three isolates sequenced here, plus 104 genomes downloaded from NCBI; Table S1) using kSNP3 version 3.1 [26] and the optimal *k*-mer size determined by Kchooser (*k* = 19). The maximum parsimony tree produced by kSNP3 was used to cluster the genomes on the basis of the core SNPs identified. 

The CFSAN SNP Pipeline was used to identify high-quality SNPs (hqSNPs) using trimmed Illumina reads associated with two different clusters, the first with the 28 SM-LI *(Salmonella* Minnesota lineage I) isolates and the second with the 44 SM-LII (*Salmonella* Minnesota lineage II) isolates. The genome assembly of isolate SRR1646144_UK_2012 was used as reference (i.e., assembly with N50 > 100,000 nt and <50 contigs). The resulting SNP matrix of preserved sites was used to build a phylogeny using the maximum likelihood (ML) method implemented in W-IQ-TREE (the IQ-TREE web server; accessed 11 December 2019) [27], using ModelFinder to select the optimal substitution model [28] and 1000 replicates of the UltraFast bootstrap approximation [29].

### 2.5. Tip-Dated Evolutionary Analysis

The linear regression approach implemented in TempEst version 1.5 [30] was used to evaluate the temporal signal and clock-likeness of the ML phylogenies constructed using hqSNPs detected among the SM-LI and SM-LII genomes. The resulting *R*^2^ value produced by TempEst was 0.45 for SM-LI and 0.53 for SM-LII. Thus, these two separate datasets were queried individually in subsequent temporal analyses.

A tip-dated phylogeny was constructed using BEAUti version 1.8.2 and BEAST version 1.8.2 [31], using combinations of the general time reversible (GTR) model [32] and one of (i) a strict clock and coalescent constant size population model, (ii) a strict clock and coalescent Bayesian skyline model, (iii) a lognormal relaxed clock and coalescent constant size population model, and (iv) a lognormal relaxed clock and coalescent Bayesian skyline model, for multiple datasets including SM-LI and SM-LII. For all models, the initial clock rate was set to 2.1 × 10^−7^ substitutions/site/year.

The Markov chain Monte Carlo (MCMC) algorithm implemented in BEAST was run for 1 × 10^9^ generations, and parameters were logged every 1 × 10^5^ generations. The optimal model (i.e., the strict clock and coalescent Bayesian skyline model) was identified using marginal likelihood estimates obtained via path-sampling using 10 steps of at least 1 × 10^9^ generations, and by assessing effective sample size (ESS) values and mixing of parameters in Tracer version 1.6.0 [33]. Five independent MCMC runs using the optimal model were performed, using chain lengths of 1 × 10^9^ generations, sampling every 1 × 10^5^ generations. The resulting log files were viewed in Tracer to ensure that ESS values were sufficiently high (i.e., >200 for all parameters) and that all parameters had mixed adequately with 10% burn-in. LogCombiner version 1.8.3 was used to combine the log and tree files of five independent runs, and TreeAnnotator version 1.8.2 [34] was used to construct a maximum clade credibility (MCC) tree, using 10% burn-in and common ancestor node heights. FigTree version 1.4.2 (http://tree.bio.ed.ac.uk/software/figtree/) was used to annotate the resulting phylogeny, using bars to denote 95% highest posterior density (HPD) intervals for node heights and branch labels to denote posterior probabilities.

### 2.6. Identification of Clade-Associated Genes within S. Minnesota 

Roary version 3.12.0 [35] was used to identify orthologous genes present in the *S.* Minnesota core and pan genome, using a minimum protein BLAST (BLASTP) identity value of 90% (-i 90). Scoary version 1.6.14 [36] was used to identify genes associated with each of two clades (i.e., SM-PLI (*Salmonella* Minnesota poultry lineage I) and SM-PLII (*Salmonella* Minnesota poultry lineage II)) within the *S*. Minnesota lineages, using a *p*-value cutoff of 0.05. 

## 3. Results 

### 3.1. WGS Data of S. Minnesota and Sequence Types (STs) 

Raw sequencing reads obtained in this study and downloaded from NCBI (Table S1) were assembled with a median of 67 contigs larger than 1 kb (ranging from 28 to 457 kb), a median N50 of 227,625 bp (ranging from 21,874 to 429,939 bp), and median average coverage of 70× (ranging from 20× to 345×). The median length of the 107 assembled *S.* Minnesota genomes (made of contigs > 1000 bp) was 4.81 Mbp (ranging from 4.49 to 5.18 Mbp). In silico analysis showed that 102 sequences matched to ST 548 and five sequences to ST 285 (Table S1). The difference between ST 548 and ST 285 was in one single-nucleotide polymorphism of the *hisD* gene.

### 3.2. S. Minnesota Isolates of Brazilian Origin Are Confined to Two Lineages

**Figure 1:**
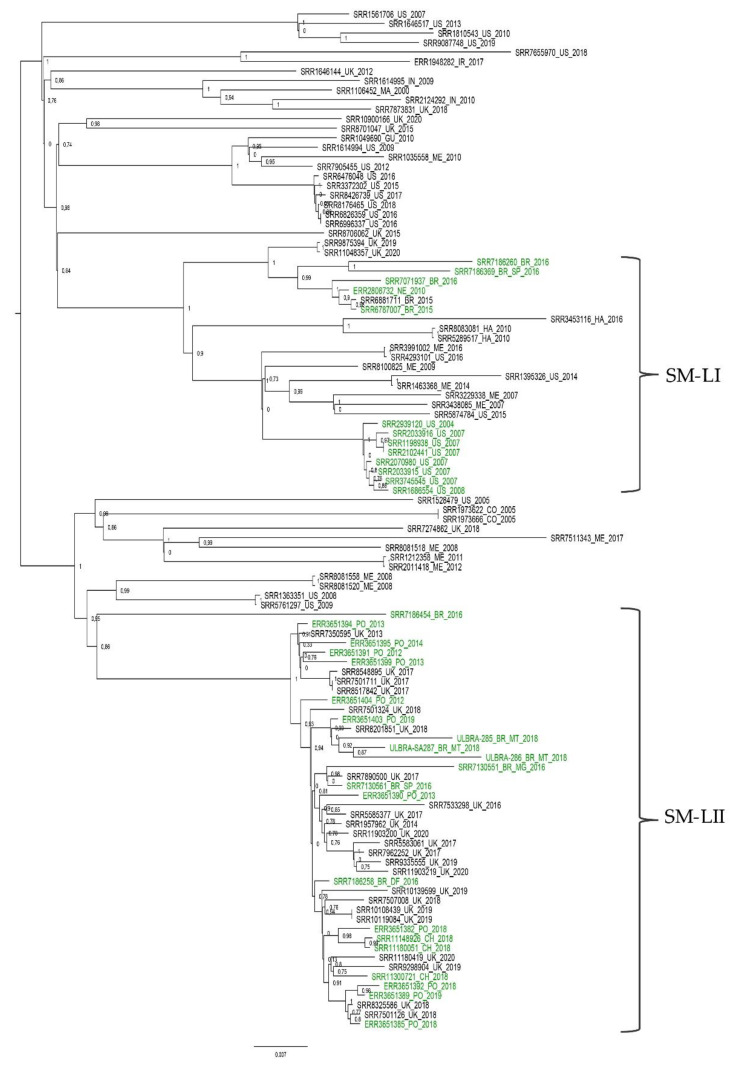
Maximum parsimony phylogeny construct using core single-nucleotide polymorphisms (SNPs) identified among 107 *Salmonella* Minnesota genomes using kSNP3. All Brazilian *S.* Minnesota isolates were grouped into two clades (*Salmonella* Minnesota lineages I and II (SM-LI and SM-LII)), denoted using Roman numerals. The label colors denote the source of isolates (green for poultry genomes). The phylogeny constructed using the maximum parsimony method implemented in kSNP3 is midpoint rooted, and branches withbootstrap values are labeled.

A maximum parsimony phylogeny constructed using core SNPs (*n* = 39,752) identified among all 107 *S.* Minnesota genomes revealed the distribution of all sequences in several clusters with no strong relationship to specific countries and sources (poultry, human, environment, food, animal feed, and livestock). According to this phylogeny, Brazilian isolates were grouped into two major specific clusters, here called *Salmonella* Minnesota lineages I and II (SM-LI and SM-LII) (Figure 1).

**Figure 2:**
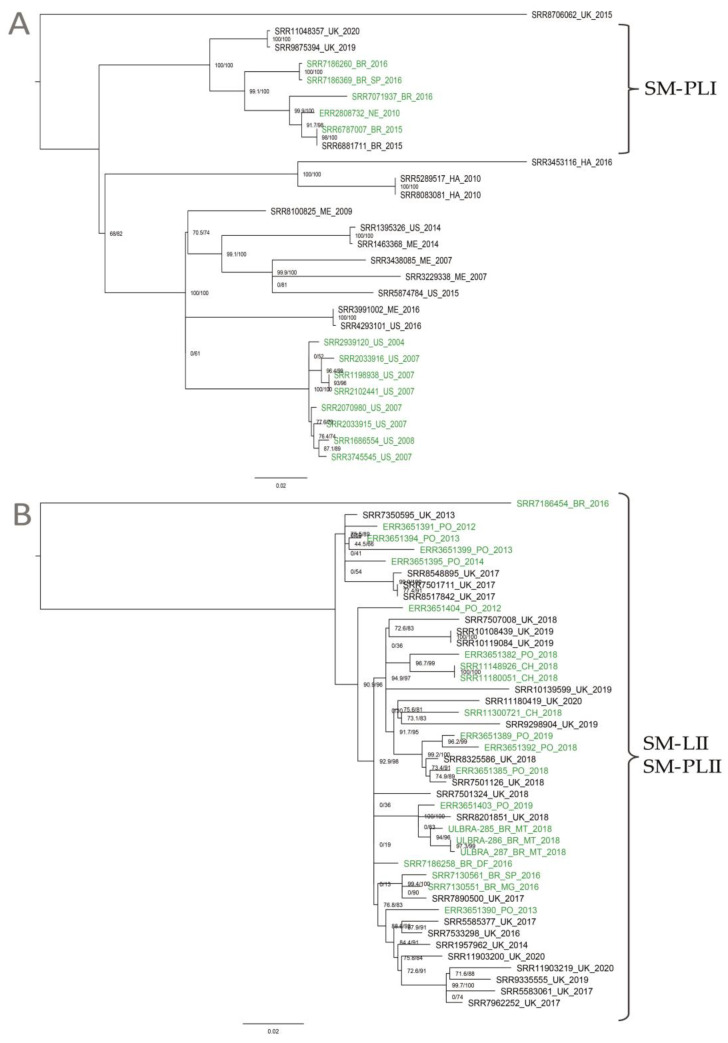
Maximum likelihood phylogeny constructed using high-quality SNPs (hqSNPs) identified among 28 SM-LI and 44 SM-LII genomes using the CFSAN SNP Pipeline. The label colors denote the source of isolates (green for poultry). The phylogeny was constructed using IQ-TREE and is midpoint rooted, with branch lengths representing the number of substitutions per site, and the bootstrap values are labeled. (**A**) 28 SM-LI genomes. In clade SM-PLI (*n* = 8) are five Brazilian isolates, two from the United Kingdom, and one from Netherlands, deposited in the National Center for Biotechnology Information (NCBI). (**B**) 44 SM-LII genomes. In clade SM-PLII (*n* = 44) are the three isolates sequenced in this study, four Brazilian isolates, three isolates from Chile, 11 isolates from Portugal, and 23 isolates from the United Kingdom, deposited in the NCBI.

Phylogenomic relationships among the two clusters (SM-LI and SM-LII) were, thus, further investigated. Using the high-quality SNP (hqSNP) calling approach implemented in the CFSAN SNP Pipeline, the maximum likelihood (ML) phylogeny clearly demonstrated that Brazilian genomes clustered into SM-LI and SM-LII. SM-LI included a total of 28 isolates: 11 from the United States, five from Brazil, five from Mexico, three from the United Kingdom, three from Haiti, and one from the Netherlands. This lineage differed by 0–322 pairwise hqSNPs (median 160) and included *S.* Minnesota isolates from four different sources: poultry, food, human, and the environment. A subclade within this lineage, referred to here as SM-PLI, contained eight genomes, including five from Brazil, two from the United States, and one from the Netherlands, and differed by 0–74 pairwise hqSNPs (median 50). These isolates were derived from different sources, including five from poultry, two from foods, and one from human feces (Figure 2A). SM-LII included 44 isolates: 23 from the United Kingdom, 11 from the Portugal, seven from Brazil, and three from Chile. This second lineage differed by 0–202 pairwise hqSNPs (median 37) and included isolates from three different sources: 21 from poultry, 20 from food, and three from humans. This whole clade is referred to hereafter as SM-PLII, since it included mainly poultry isolates (Figure 2B). 

### 3.3. Bayesian Phylogenetic Analysis of S. Minnesota

**Figure 3:**
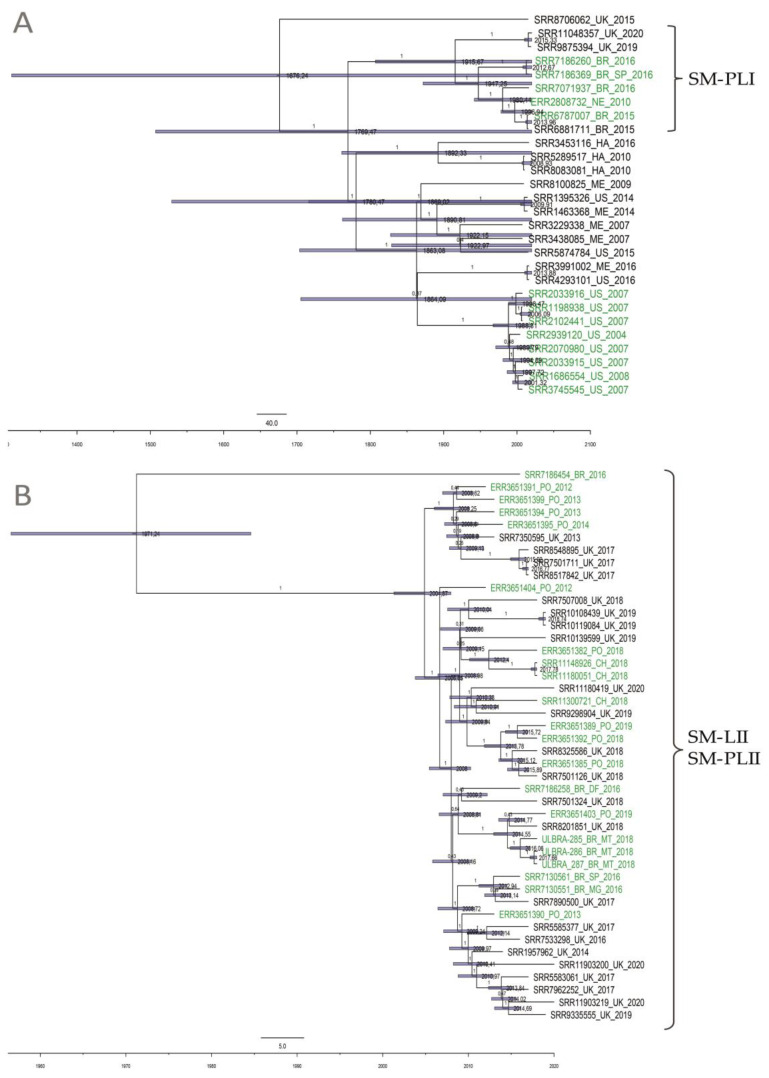
Maximum clade credibility tree constructed using high-quality SNPs (hqSNPs) identified among two *S.* Minnesota lineages using the CFSAN SNP Pipeline, rooted using BEAST. Time in years is plotted along the *x*-axis. Branch labels denote posterior probabilities of branch support, and node bars correspond to 95% highest posterior density (HPD) intervals for node heights. The label colors denote the source of isolates (green for poultry genomes). (**A**) 28 SM-LI genomes shared a common ancestor emerging in the year 1676. Clade SM-PLI shared a common ancestor that existed circa 1915. (**B**) 44 SM-PLII genomes shared a common ancestor emerging in the year 1971.

The evolutionary rate for the SM-LI isolates was predicted to be 1.27 × 10^−7^ substitutions/site/year (95% HPD 1.96 × 10^−8^–2.39 × 10^−7^) and shared a common ancestor emerging in the year 1676 (95% HPD 928–1765). SM-PLI shared a common ancestor that existed circa 1915 (95% HPD 1375–1982) (Figure 3A). The evolutionary rate for the clade SM-PLII was predicted to be 4.29 × 10^−7^ substitutions/site/year (95% HPD 3.06 × 10^−7^–5.49 × 10^−7^) and shared a common ancestor emerging in 1971 (95% HPD 1908–2006). Most SM-PLII isolates (97%; 43/44) shared a common ancestor emerging even more recently in 2004 (95% HPD 1986–2016), suggesting the high dissemination of this lineage in the beginning of the 2000s (Figure 3B). 

**Figure 4:**
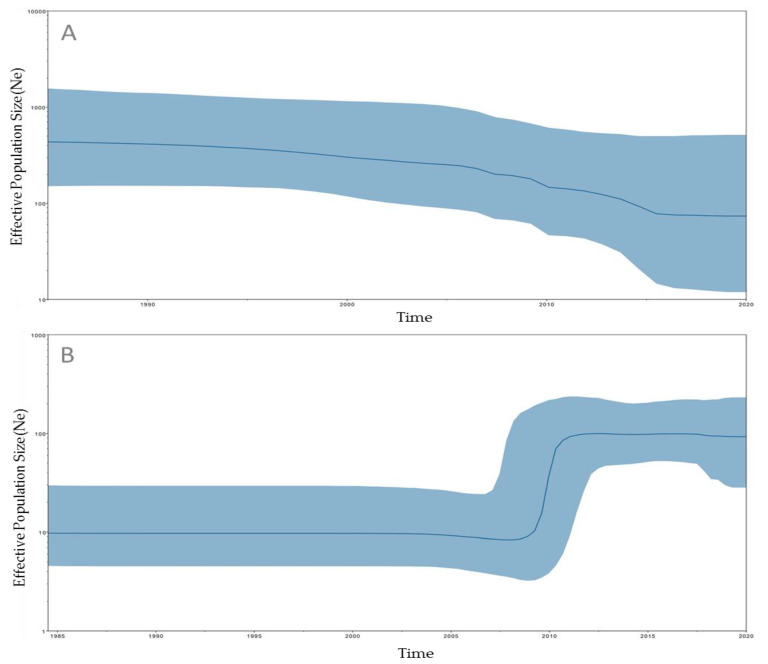
Bayesian skyline plot constructed via BEAST and Tracer, using high-quality SNPs (hqSNPs) detected among SM-LI and SM-LII/SM-PLII. The *x*-axis represents time in years, while the *y*-axis denotes the effective population size. The dark-blue line represents the median, while light-blue shading represents the 95% highest posterior density (HPD) interval. (**A**) Bayesian skyline plot detected among 28 SM-LI genomes. (**B**) Bayesian skyline plot detected among 44 SM-LII/SM-PLII genomes.

Historical demographic trends of the median estimate of the *S.* Minnesota effective population sizes of SM-LI and SM-PLII over time were reconstructed using a Bayesian skyline approach. The SM-LI effective population size remained constant from 1985 to 2000 (Figure 4A). The SM-PLII effective population size remained constant from 1985 until 2009, when there was an increase until 2012, after which the effective population size remained constant through 2020 (Figure 4B).

### 3.4. Genes That Confer Resistance to Tetracyclines and Sulfonamides Are Prevalent among the S. Minnesota Poultry Lineages

Of the 107 *S*. Minnesota genomes queried here, 11.2% (12/107) did not have plasmid replicons, while 88.8% (95/107) possessed one to six replicons. The following replicons were detected (in order of frequency): ColRNAI (*n* = 61; 57.1%), IncA/C2 (*n* = 47; 43.9%), IncFIC(FII) (*n* = 23; 21.5%), ColpVC (*n* = 15; 14.1%), and IncFII(S) (*n* = 11; 10.2%). Plasmid replicons Col(MGD2), Col(Ye449), Col156, Col8282, IncFIB(AP001918), IncFIB(pB171), IncFIB(pECLA), IncFIB(pHCM2), IncFIB(29), IncFII(SARC14), IncFII(p14), IncFII(p96A), IncFII(pECLA), IncFII(pHN7A8), IncFII(pKPX1), IncFII(pRSB107), IncFII, IncFII_1_pSFO, IncI1, IncY, and pSL483 were each detected among 107 genomes, but in fewer than 10 isolates each (i.e., with frequencies less than 10%). 

A separate analysis of the eight SM-PLI genomes demonstrated the occurrence of six different plasmid replicons. One SM-PLI sequence (12.5%) did not have plasmid replicons, while the remaining seven (87.5%) possessed one to three of them. The following replicons were detected most frequently: IncA/C2 (*n* = 4; 50%) and ColRNAI (*n* = 3; 37.5%) (Figure S1). Among the 44 SM-PLII genomes, 13 different plasmid replicons were detected, and each isolate genome harbored one to six of these replicons. The following replicons were detected most frequently: IncA/C2 (*n* = 43; 97.7%) and ColRNAI (*n* = 40; 90.1%) (Figure S1).

Twenty-eight different antimicrobial resistance genes were detected among 107 isolates, with each genome harboring 2–11 genes and/or integrons. The *aac(6′)-Iaa* and *mdf(A)* genes, which confer resistance to amynoglicosides (*aac(6′)-Iaa*), benzalkonium chloride (*mdf(A)*), and rhodamine (*mdf(A)*), were detected in all 107 genomes (100%). The *sul2, tet(A)*, *blaCMY-2, aph(3′)-Ia_1*, *qnrB19,* and *ant(3′’)*-Ia genes, which confer resistance to sulfonamides, tetracyclines, cephamycins, cephalosporins, aminoglycoside phosphotransferases, fluoroquinolones, and streptomycins, respectively, were less frequent (*n* = 55 (51.4%), *n* = 48 (44.6%), *n* = 42 (39.2%), *n* = 39 (36.5%), *n* = 38 (35.5%), *n* = 25 (23.3%), respectively). The remaining 20 genes, which confer resistance to aminoglycosides, cephalosporins, diaminopyrimidines, penams, penems, phosphomycins, phenicols, tetracycline, and sulfonamides, were detected in fewer than 8% of the isolates.

Among the eight SM-PLI genomes, 12 different antimicrobial resistance genes and/or integrons were detected. Apart from genes detected in 100% of the isolates (i.e., *aac(6′)-Iaa* and *mdf(A)*), the following other antimicrobial resistance genes were present: *sul2* and *tet(A)* in four isolates (50%) each (Figure S1, Supplementary Materials). In addition, 13 different antimicrobial resistance genes and/or integrons were detected among the 44 SM-PLII genomes. Apart from genes detected in 100% of the isolates (i.e., *aac(6′)-Iaa* and *mdf(A)*), other antimicrobial resistance genes included *sul2* and *tet(A)* in 43 isolates (97.7%), *blaCMY-2* in 42 isolates (95.4%), *aph(3′)-Ia* and *qnrB19* in 28 isolates (86.3%), and *ant(3′’)-Ia* in 23 isolates (52.7%) (Figure S1).

### 3.5. Virulence Mapping and Pan-Genome of S. Minnesota 

To understand the pathogenicity repertoire of *S.* Minnesota isolates, genetic virulence factors were queried in the 107 genomes. Four pathogenicity islands (SPI2, SPI5, SPI13, and SPI14) were detected in all isolates, two (SPI3 and SPI9) were detected in 106 isolates (99.1%), two (SPI4 and SPI1) were detected in 103 isolates (96.2%), and C63PI was detected in 99 isolates (92.5%). A separate analysis of the eight SM-PLI genomes demonstrated that seven pathogenicity islands (SPI2, SPI3, SPI4, SPI5, SPI9, SPI13, and SPI14) were detected in all isolates, C63PI was detected in seven isolates (87.5%), and SPI1 was detected in five isolates (62.5%). Among the 44 SM-PLII genomes, seven pathogenicity islands (SPI1, SPI2, SPI5, SPI9, SPI13, SPI14, and C63PI) were detected in all isolates, SPI3 was detected in 43 isolates (97.2%), and SPI4 was detected in 40 isolates (90.9%). Overall, 160 *Salmonella* virulence genes were detected, with 113 genes present in all isolates. Noteworthy, there was the additional occurrence of some specific virulence genes in Brazilian lineages; 50% SM-PLI genomes (4/8) and 93.1% SM-PLII (41/44) genomes carried *fyuA*, *irp1*, *irp2* and the operon *ybtABPQSTUX* (Figure S2) with a median of 100% identity and 97% coverage.

Of the 15,320 genes identified among the 107 *S*. Minnesota genomes, 3873 (25.2%) were core genes (i.e., present among all 107 genomes), while the remaining 11,447 (74.8%) comprised the pan-genome (Figure S3).

## 4. Discussion

*S.* Minnesota is a foodborne pathogen recently associated with the Brazilian poultry production chain. This serotype has been increasingly detected in avian farms from the Center West, one of the fastest growing poultry-producing regions in Brazil [6]. In this study, three *S.* Minnesota strains from this region were isolated and sequenced and compared to other genomes submitted to NCBI’s SRA database as *S.* Minnesota (*n* = 104), including nine more from Brazil.

In order to study the phylogenetic relationships of Brazilian *S.* Minnesota isolates of poultry origin, all 107 complete genomes were first assigned to STs using seven-gene MLST. Only STs 548 and 285 were detected, and the former was more frequent, as previously reported [7,37]. In the phylogeny (Figure 1), ST 285 was nested within ST 548. ST 285 is rare and normally detected in Mexico, although some strains have been detected in the United Kingdom and the United States from environment, livestock, human and avian sources (http://enterobase.warwick.ac.uk/species/senterica/search_strains?query=st_search). 

Despite encompassing two STs, the *S.* Minnesota genomes queried here displayed considerable genomic diversity, and Brazilian isolates clustered within two specific clades (SM-LI and SM-LII). Additionally, it could be observed that some European *S.* Minnesota genomes also clustered with SM-PLI and SM-PLII. Noteworthy, the occurrence of *S*. Minnesota isolates in Europe has already been previously described, including in poultry meat imported from Brazil [8,11]. A more recent study also associated some *S*. Minnesota isolates (*n* = 3) with poultry production in Brazil [37]. These previously sequenced *S.* Minnesota genomes from Brazil clustered in one unique clade together with isolates from the United Kingdom, all being predominantly found in humans and food, according to a core genome phylogeny [37]. Furthermore, a temporal study of *Salmonella enterica* serotypes from broiler farms in Brazil showed that *S*. Minnesota was the most frequent serotype in broiler flocks, and the transmission on the surveyed farms was predominantly horizontal and through different contamination sources [6].

The Bayesian temporal phylogenetic evaluation showed that the common ancestor of the SM-PLI and SM-PLII presented tMRCAs in the 20th century (around 1915 and 1971, respectively). Interestingly, 97.7% (43/44) SM-PLII isolates shared a common ancestor that existed circa 2004, and a recent increase in effective population size was also estimated (mainly from 2009 to 2012; Figure 4B). There are too few isolates from this lineage associated with poultry production in the specific Brazilian producing regions to conclusively say that *S.* Minnesota is spreading due to SM-PLII in Brazil. Further studies should be carried out with more *S.* Minnesota genomes from poultry sources to reach at more detailed information about the main lineages and their temporal evolution in this specific animal production chain. However, the three isolates sequenced here, and other information obtained from field reports demonstrated the high recent dissemination of this serotype, probably related to the recent expansion of poultry production in Brazil in the last two decades [38,39]. The increased global trade of poultry meat and recent major changes in international microbiological food regulations have further evidenced the occurrence of this *Salmonella* serotype in chicken meat and other poultry products [11,40,41].

*S.* Minnesota isolates sequenced here and others from poultry were predicted to be resistant to multiple antibiotics and to carry genes *ant(3′’)-Ia, aph(3′)-Ia, blaCMY-2, mdf(A), qnrB19, sul2*, and *tet(A)*, which confer resistance to the class of aminoglycosides, betalactams, fluoroquinolones, sulfonamides, and tetracyclines. These genes and even a multidrug resistance (MDR) profile have already been described in other studies of *S.* Minnesota [11,37,40]. This gene set was probably selected as a result of management practices in the Brazilian poultry production chain. A recent study reported several places in Brazil as hotspots of antimicrobial resistance emergence [41].

Most SM-PLI and SM-PLII isolates also possessed some recognized virulence genes, such as *fyuA*, *irp1*, *irp2*, and the operon *ybtAPQSTUX* (i.e., median of 100% identity and 97% coverage). This last gene cluster is usually located in a chromosomal region called the highly pathogenic island (HPI). HPI encodes a yersiniabactin-mediated iron acquisition system previously demonstrated in pathogenic strains of *Yersinia* and several members of the Enterobacteriaceae, including *Salmonella* [42]. HPI has been reported in other *Salmonella* serotypes, such as Infantis [43] and Seftenberg [42]; however, to our knowledge, this is the first study identifying HPI in *S.* Minnesota. The HPI presence in *S.* Minnesota could provide a more concerning metabolic profile in different ways: (i) improving the environmental fitness and persistence of the bacteria [44], (ii) suppressing the host immune response [45], and (iii) influencing the expression of virulence determinants or additional genes not associated with this specific island, which may increase their ability to initiate infection [44]. These additional genetic properties may therefore, be, determinants for the dissemination and persistence of *S.* Minnesota in Brazilian poultry farms [6]. New studies would be necessary to experimentally confirm this genomic evidence.

## 5. Conclusions

The present study provided insights into the evolution and genomic content of *S.* Minnesota, particularly the lineages currently circulating in the poultry production chain in Brazil. Two specific lineages (SM-PLI and SM-PLII) were identified, as well as numerous isolates that possessed genes conferring antibiotic resistance and high genetic virulence potential. There are still few studies in the literature evaluating this important *Salmonella* serotype, and there were no data on the evolutionary analysis of *S.* Minnesota lineages. This information can contribute to the improvement of the management of all production systems affected by this serotype, particularly avian flocks in the poultry production chain from Brazil.

## Supplementary Materials

The following are available online at https://www.mdpi.com/2076-2615/10/11/2043/s1: Table S1. Metadata of *S*. Minnesota isolates from NCBI and three isolates sequenced in this study; Figure S1. Presence and absence of 28 antimicrobial resistance genes and 26 plasmids replicons among 107 *S.* Minnesota genomes; Figure S2. Presence and absence of 47 virulence genes among 107 *S.* Minnesota genomes; Figure S3. Pie plots of gene content in core, soft core, shell, and cloud genomes describing the pan genome for *S*. Minnesota.
