# Supplementary material for: Genomic Characterization of Salmonella Minnesota Clonal Lineages Associated with Poultry Production in Brazil
Source: Animals (Basel). 2020 Nov 5;10(11):2043. doi: 10.3390/ani10112043 (PMC7694379; doi:10.3390/ani10112043)
Supplement: Supplementary file 1 [file animals-10-02043-s001.pdf]

animals

MDPI

Supplementary Materials: Genomic Characterization of *Salmonella* Minnesota Clonal Lineages Associated with Poultry Production in Brazil

Diéssy Kipper [1], Laura M. Carroll [2], Andrea K. Mascitti [1], André F. Streck [3], André S.K. Fonseca [4], Nilo Ikuta [4] and Vagner R. Lunge [1,4,*]

[1] Laboratório de Diagnóstico Molecular, Universidade Luterana do Brasil (ULBRA), Canoas, Rio Grande do Sul, 92425-020, Brazil; diessykipper@hotmail.com (D.K.); andreakaroline88@hotmail.com (A.K.M.)
[2] Department of Food Science, Cornell University, Ithaca, New York, NY, 14850, USA; laura.carroll@embl.de
[3] Laboratório de Diagnóstico em Medicina Veterinária, Universidade de Caxias do Sul (UCS), Caxias do Sul, Rio Grande do Sul, 95070-560, Brazil; afstreck@ucs.br
[4] Simbios Biotecnologia, Cachoeirinha, Rio Grande do Sul, 94940-030, Brazil; fonseca@simbios.com.br (A.S.K.F.); ikuta@simbios.com.br (N.I.)
* Correspondence: vagner.lunge@gmail.com; Tel.: +55-5199984-1770

Table S1. Metadata of *S*. Minnesota isolates from NCBI and three isolates sequenced in this study.

Lineage	Identification	SRA accession	Date	Country	Source	ST	Contigs	N50 (bp)	Length	Average Coverage
SM-LI	SRR4293101_US_2016	SRR4293101	2016	US	Blood (Human)	548	36	429933	4534716	108
SM-LI	SRR3991002_ME_2016	SRR3991002	2016	ME	Cantaloupe (Environment)	548	52	429933	4544362	103
SM-LI	SRR8083081_HA_2010	SRR8083081	2010	HA	Dried coconut (Environment)	548	71	142196	4735224	49
SM-LI	SRR5289517_HA_2010	SRR5289517	2010	HA	Dried coconut (Environment)	548	457	62494	5169870	69
SM-LI	SRR3229338_ME_2007	SRR3229338	2007	ME	Abandgo tea (Food)	548	125	230716	5044648	123
SM-LI	SRR3453116_HA_2016	SRR3453116	2016	HA	Fresh thai chilli pepper (Food)	548	181	52207	4808212	68
SM-LI	SRR3438085_ME_2007	SRR3438085	2007	ME	Gincoforte capsules (Food)	548	110	150796	5040508	109
SM-LI	SRR2939120_US_2004	SRR2939120	2004	US	Ground turkey (Poultry)	548	28	415980	4689809	89
SM-LI	SRR2102441_US_2007	SRR2102441	2007	US	Ground turkey (Poultry)	548	45	227740	4718737	80
SM-LI	SRR1686554_US_2008	SRR1686554	2008	US	Ground turkey (Poultry)	548	41	242559	4657454	101
SM-LI	SRR8706062_UK_2015	SRR8706062	2015	UK	Human	548	36	276351	4604628	62
SM-LI	SRR1463368_ME_2014	SRR1463368	2014	ME	Mango (Environment)	548	103	111078	4929951	149
SM-LI	SRR1395326_US_2014	SRR1395326	2014	US	Mango (Environment)	548	87	225303	4939124	76
SM-LI	SRR1198938_US_2007	SRR1198938	2007	US	Poultry	548	75	275337	4730766	56
SM-LI	SRR2033915_US_2007	SRR2033915	2007	US	Poultry	548	72	274984	4659118	64
SM-LI	SRR2033916_US_2007	SRR2033916	2007	US	Poultry	548	104	374343	4811396	65
SM-LI	SRR2070980_US_2007	SRR2070980	2007	US	Poultry	548	81	265080	4709943	93
SM-LI	SRR3745545_US_2007	SRR3745545	2007	US	Poultry	548	84	265017	4673281	70
SM-LI	SRR8100825_ME_2009	SRR8100825	2009	ME	River water (Environment)	548	130	141573	4890555	35
SM-LI	SRR5874784_US_2015	SRR5874784	2015	US	Stool (Human)	548	99	373456	5188842	89
SM-LI / SM-PLI	SRR6787007_BR_2015	SRR6787007	2015	BR	Broiler chicken (Poultry)	548	103	118415	5016449	67
SM-LI / SM-PLI	ERR2808732_NE_2010	ERR2808732	2010	NE	Broiler chicken (Poultry)	548	74	133398	4838032	108
SM-LI / SM-PLI	SRR11048357_UK_2020	SRR11048357	2020	UK	Food	548	52	228686	4668500	56
SM-LI / SM-PLI	SRR9875394_UK_2019	SRR9875394	2019	UK	Human	548	39	229288	4664415	89
SM-LI / SM-PLI	SRR7071937_BR_2016	SRR7071937	2016	BR	Mechanically recovered meat (Poultry)	548	85	122131	4752994	55

SM-LI / SM-PLI	SRR7186369_BR_SP_2016	SRR7186369	2016	BR	Mechanically recovered meat (Poultry)	548	48	225304	4570280	70
SM-LI / SM-PLI	SRR7186260_BR_2016	SRR7186260	2016	BR	Mechanically recovered meat (Poultry)	548	42	268951	4572177	56
SM-LI / SM-PLI	SRR6881711_BR_2015	SRR6881711	2015	BR	Broiler chicken (Poultry)	548	107	110921	4840173	63
SM-LII / SM-PLII	SRR7501711_UK_2017	SRR7501711	2017	UK	Food	548	48	227625	4931204	74
SM-LII / SM-PLII	SRR7130561_BR_SP_2016	SRR7130561	2016	BR	Chicken carcass (Poultry)	548	68	172413	4848016	82
SM-LII / SM-PLII	SRR7130551_BR_MG_2016	SRR7130551	2016	BR	Chicken feet (Poultry)	548	79	136690	4893005	71
SM-LII / SM-PLII	SRR7186258_BR_DF_2016	SRR7186258	2016	BR	Chiken carcass (Poultry)	548	57	268591	4865616	66
SM-LII / SM-PLII	SRR8517842_UK_2017	SRR8517842	2017	UK	Food	548	35	265143	4838537	72
SM-LII / SM-PLII	SRR7890500_UK_2017	SRR7890500	2017	UK	Food	548	46	197516	4745633	66
SM-LII / SM-PLII	SRR7962252_UK_2017	SRR7962252	2017	UK	Food	548	63	226991	4897078	40
SM-LII / SM-PLII	SRR8201851_UK_2018	SRR8201851	2018	UK	Food	548	58	172405	4906577	85
SM-LII / SM-PLII	SRR9335555_UK_2019	SRR9335555	2019	UK	Food	548	67	262915	5029922	82
SM-LII / SM-PLII	SRR11148926_CH_2018	SRR11148926	2018	CH	Food (Poultry)	548	88	209163	4864648	53
SM-LII / SM-PLII	SRR11180051_CH_2018	SRR11180051	2018	CH	Food (Poultry)	548	105	157899	4864583	57
SM-LII / SM-PLII	SRR11300721_CH_2018	SRR11300721	2018	CH	Food (Poultry)	548	111	268590	4887702	84
SM-LII / SM-PLII	SRR10108439_UK_2019	SRR10108439	2019	UK	Food	548	59	228096	4723424	64
SM-LII / SM-PLII	SRR10139599_UK_2019	SRR10139599	2019	UK	Food	548	56	201911	4859533	134
SM-LII / SM-PLII	SRR11180419_UK_2020	SRR11180419	2020	UK	Food	548	68	163560	4855614	49
SM-LII / SM-PLII	SRR11903200_UK_2020	SRR11903200	2020	UK	Food	548	74	170217	4860345	70
SM-LII / SM-PLII	SRR11903219_UK_2020	SRR11903219	2020	UK	Food	548	81	185452	4942860	171
SM-LII / SM-PLII	SRR5583061_UK_2017	SRR5583061	2017	UK	Food	548	155	74960	4908188	38
SM-LII / SM-PLII	SRR5585377_UK_2017	SRR5585377	2017	UK	Food	548	61	179728	4819176	86
SM-LII / SM-PLII	SRR7501324_UK_2018	SRR7501324	2018	UK	Food	548	53	228013	4813269	40
SM-LII / SM-PLII	SRR7507008_UK_2018	SRR7507008	2018	UK	Food	548	81	226038	4877084	106
SM-LII / SM-PLII	SRR8325586_UK_2018	SRR8325586	2018	UK	Food	548	57	228110	4811146	78
SM-LII / SM-PLII	SRR8548895_UK_2017	SRR8548895	2017	UK	Food	548	50	225304	4841601	61
SM-LII / SM-PLII	SRR9298904_UK_2019	SRR9298904	2019	UK	Food	548	60	264650	4856463	88
SM-LII / SM-PLII	ERR3651389_PO_2019	ERR3651389	2019	PO	Food (Poultry)	548	138	232398	4989636	135
SM-LII / SM-PLII	ERR3651403_PO_2019	ERR3651403	2019	PO	Food (Poultry)	548	87	216086	4896009	93

SM-LII / SM-PLII	ERR3651385_PO_2018	ERR3651385	2018	PO	Food (Poultry)	548	102	320504	4799503	118
SM-LII / SM-PLII	ERR3651382_PO_2018	ERR3651382	2018	PO	Food (Poultry)	548	131	131697	4811941	68
SM-LII / SM-PLII	ERR3651390_PO_2013	ERR3651390	2013	PO	Food (Poultry)	548	92	268591	4793563	127
SM-LII / SM-PLII	ERR3651392_PO_2018	ERR3651392	2018	PO	Food (Poultry)	548	130	268591	4929438	150
SM-LII / SM-PLII	ERR3651399_PO_2013	ERR3651399	2013	PO	Food (Poultry)	548	204	157207	4968421	148
SM-LII / SM-PLII	ERR3651394_PO_2013	ERR3651394	2013	PO	Food (Poultry)	548	189	78337	4798715	73
SM-LII / SM-PLII	ERR3651395_PO_2014	ERR3651395	2014	PO	Food (Poultry)	548	181	76326	4889727	56
SM-LII / SM-PLII	ERR3651404_PO_2012	ERR3651404	2012	PO	Food (Poultry)	548	104	161716	4871210	86
SM-LII / SM-PLII	ERR3651391_PO_2012	ERR3651391	2012	PO	Food (Poultry)	548	182	174272	4805907	131
SM-LII / SM-PLII	SRR7350595_UK_2013	SRR7350595	2013	UK	Human	548	43	228620	4760226	44
SM-LII / SM-PLII	SRR1957962_UK_2014	SRR1957962	2014	UK	Human	548	43	229500	4797644	40
SM-LII / SM-PLII	SRR7533298_UK_2016	SRR7533298	2016	UK	Human	548	44	313442	4855237	75
SM-LII / SM-PLII	*ULBRA_285_BR_MT_2018	SRR8508322	2018	BR	Poultry	548	94	118336	4836280	101
SM-LII / SM-PLII	*ULBRA_286_BR_MT_2018	SRR8508321	2018	BR	Poultry	548	146	72948	4901034	70
SM-LII / SM-PLII	*ULBRA_287_BR_MT_2018	SRR8543997	2018	BR	Poultry	548	107	100667	4835851	60
SM-LII / SM-PLII	SRR7186454_BR_2016	SRR7186454	2016	BR	Slaughterhouse (Poultry)	548	43	268766	4533550	57
SM-LII / SM-PLII	SRR7501126_UK_2018	SRR7501126	2018	UK	Food	548	58	199812	4811742	54
SM-LII / SM-PLII	SRR10119084_UK_2019	SRR10119084	2019	UK	Food	548	64	209886	4817287	69
OL	SRR1810543_US_2010	SRR1810543	2010	US	Animal feed	548	457	21874	4561198	134
OL	SRR1561706_US_2007	SRR1561706	2007	US	Bovine feces (Livestock)	548	52	275343	4624450	205
OL	SRR1973666_CO_2005	SRR1973666	2005	CO	Cheese (Food)	548	32	392934	45554349	42
OL	SRR7655970_US_2018	SRR7655970	2018	US	Chilli Powder (Food)	548	64	243655	4784568	76
OL	SRR1035558_ME_2010	SRR1035558	2010	ME	Coconut strips (Environment)	548	107	102292	4740530	20
OL	SRR8081558_ME_2008	SRR8081558	2008	ME	Cow (Livestock)	548	75	157236	4891778	31
OL	SRR8081518_ME_2008	SRR8081518	2008	ME	Cow (Livestock)	285	100	196036	5088142	54
OL	SRR6826359_US_2016	SRR6826359	2016	US	Dietary puppy supplement (Food)	548	55	429938	4579002	90
OL	SRR1363351_US_2008	SRR1363351	2008	US	Drag swab (Environment)	548	32	429928	4495671	115
OL	SRR1212358_ME_2011	SRR1212358	2011	ME	Environmental swab (Environment)	285	64	362995	4885121	56
OL	SRR7905455_US_2012	SRR7905455	2012	US	Environmental swab (Environment)	548	167	103401	4990242	58

OL	SRR1646517_US_2013	SRR1646517	2013	US	Environmental swab (Environment)	548	37	321012	4629312	77
OL	SRR6996337_US_2016	SRR6996337	2016	US	Finished pet food (Animal Feed)	548	41	275322	4576890	52
OL	SRR8426739_US_2017	SRR8426739	2017	US	Finished pet food (Animal feed)	548	51	218787	4564541	64
OL	SRR10900166_UK_2020	SRR10900166	2020	UK	Food	548	40	263547	4536149	111
OL	SRR8081520_ME_2008	SRR8081520	2008	ME	Goat (Food)	548	74	217079	4893819	33
OL	SRR1528479_US_2005	SRR1528479	2005	US	Ground beef (Livestock)	548	45	312908	4695006	102
OL	SRR6476048_US_2016	SRR6476048	2016	US	Ground component turkey (Poultry)	548	56	318088	4566635	82
OL	SRR1973622_CO_2005	SRR1973622	2005	CO	Ham (Food)	548	38	320830	4555321	104
OL	SRR7274862_UK_2018	SRR7274862	2018	UK	Homo sapiens (Human)	285	106	170666	5047029	39
OL	SRR1049690_GU_2010	SRR1049690	2010	GU	Hulled sesame (Environment)	548	38	429939	4528737	66
OL	ERR1948282_IR_2017	ERR1948282	2017	IR	Human	548	115	80663	4677880	32
OL	SRR1646144_UK_2012	SRR1646144	2012	UK	Human	548	36	230238	4506808	76
OL	SRR8701047_UK_2015	SRR8701047	2015	UK	Human	548	46	227001	4850346	79
OL	SRR7873831_UK_2018	SRR7873831	2018	UK	Human	548	45	227754	4729339	53
OL	SRR1106452_MA_2000	SRR1106452	2000	MA	Meat	548	42	204523	4504795	132
OL	SRR1614995_IN_2009	SRR1614995	2009	IN	Nimbu masala (Food)	548	149	153101	4630771	88
OL	SRR3372302_US_2015	SRR3372302	2015	US	Pet food (Animal feed)	548	34	429938	4506808	69
OL	SRR8176465_US_2018	SRR8176465	2018	US	Poultry	548	43	275327	4574979	40
OL	SRR9087748_US_2019	SRR9087748	2019	US	Poultry	548	111	153957	4800800	61
OL	SRR2124292_IN_2010	SRR2124292	2010	IN	Sesame seeds (Environment)	548	50	227740	4629034	44
OL	SRR1614994_US_2009	SRR1614994	2009	US	Sesame seeds (Food)	548	44	318552	4565774	66
OL	SRR5761297_US_2009	SRR5761297	2009	US	Stool (Human)	548	30	294447	4609619	52
OL	SRR2011418_ME_2012	SRR2011418	2012	ME	Surface (Environment)	285	67	268416	4880053	345
OL	SRR7511343_ME_2017	SRR7511343	2017	ME	Water (Environment)	285	102	111034	4914349	37

OL – Other Lineage; US – United States; ME – Mexico; HA – Haiti; UK – United Kingdom; BR – Brazil; NE – Netherlands; Ch – Chile; PO – Portugal; CO – Colombia; GU – Guatemala; IR – Ireland; MA – Malaysia; IN – India. * Isolates sequenced in this study.

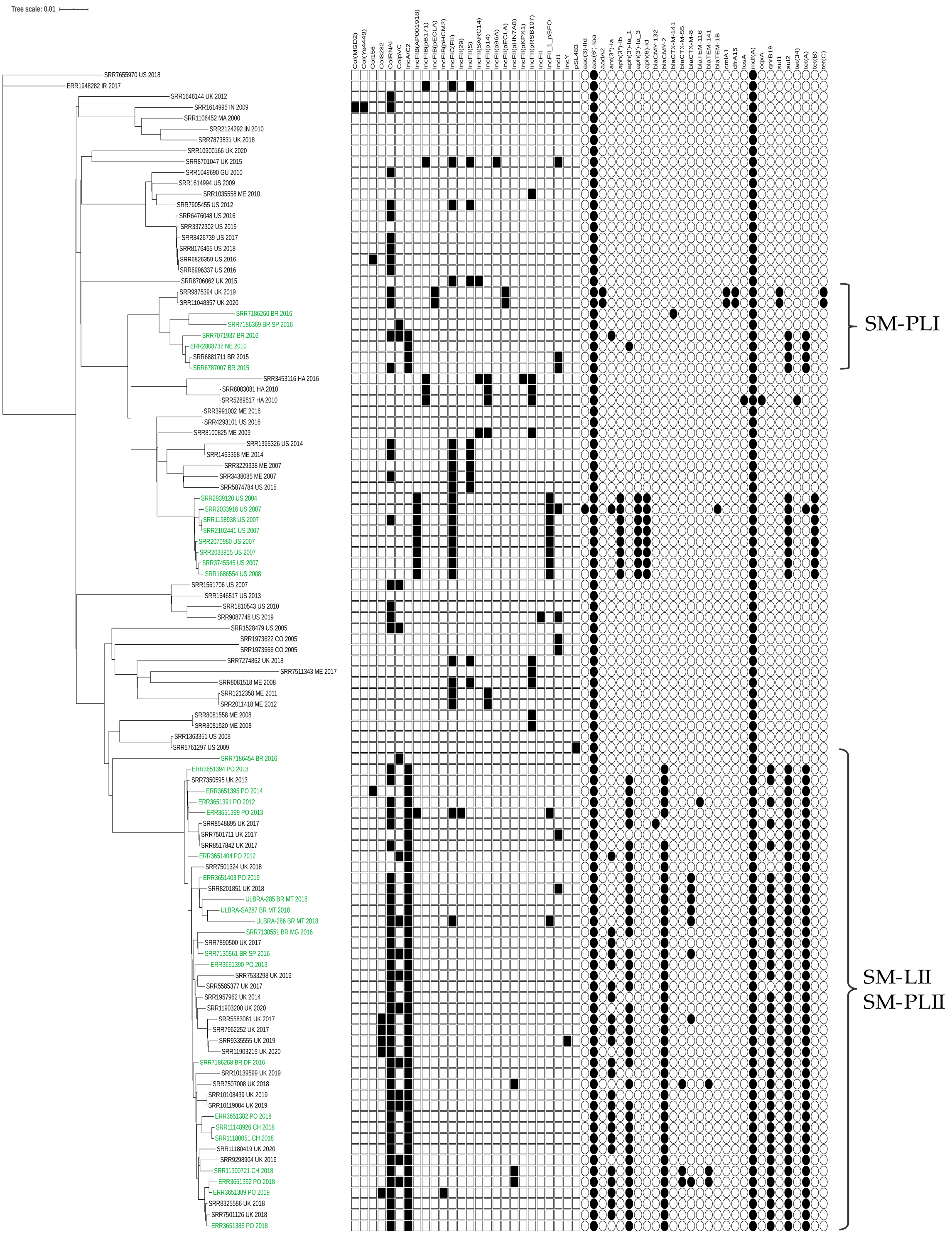

Figure S1. Presence and absence of 28 antimicrobial resistance genes and 26 plasmids replicons among 107 *S.* Minnesota genomes. Black and white squares in the heat map denote the presence and absence of a plasmid replicon, respectively. Black and white circles in the heat map denote the presence and absence of an antimicrobial resistance gene, respectively. The names of the antimicrobial resistance genes and plasmid replicons are at the top of the heatmap. Labels are at the tips of maximum parsimony method implemented in kSNP3 (Figure 1). The label colors denote the source of isolates (green for poultry genomes). iTOL was used to build this heat map.

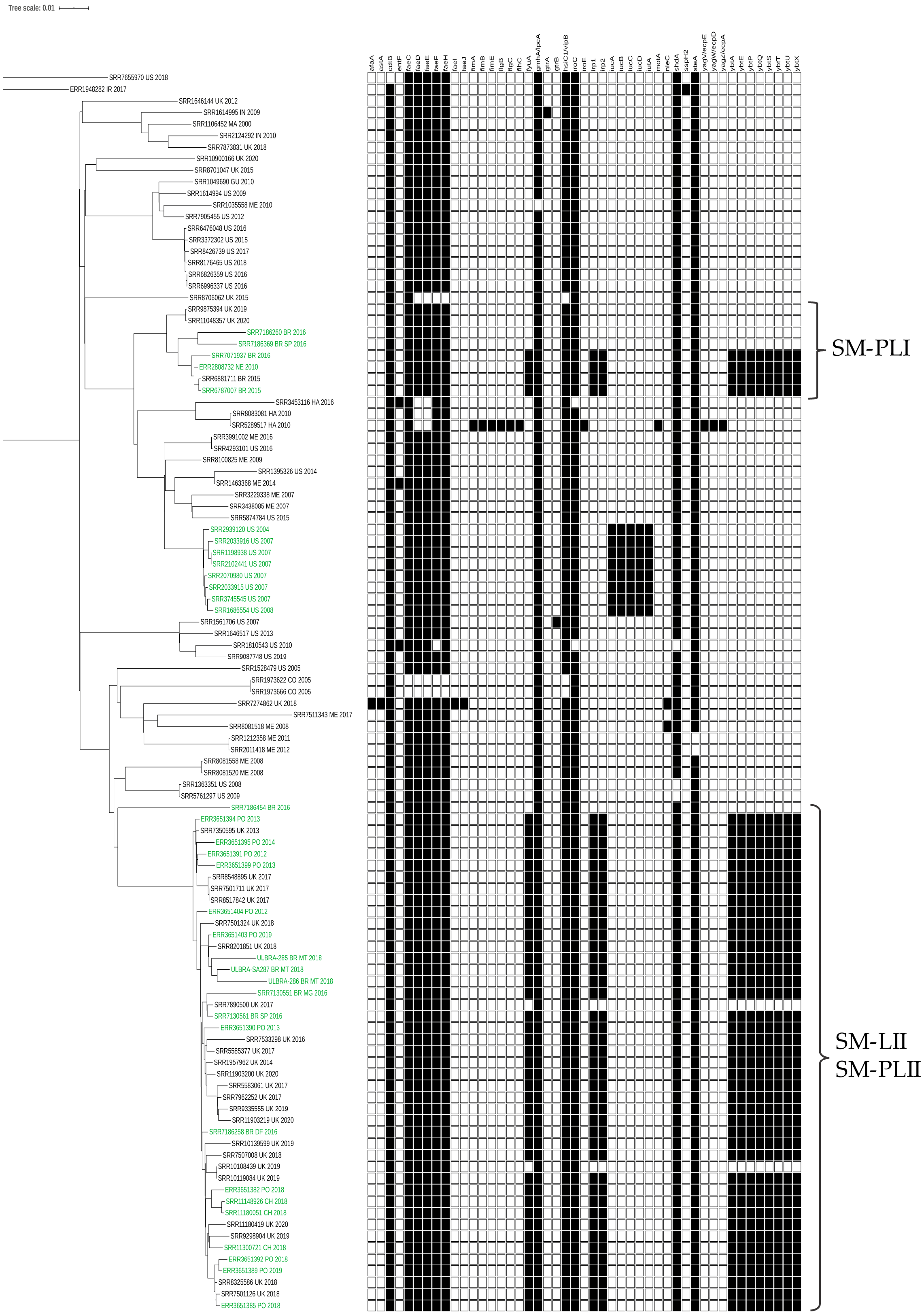

Figure S2. Presence and absence of 47 virulence genes among 107 *S.* Minnesota genomes. Black and white squares in the heat map denote the presence and absence of a virulence gene, respectively. The names of the virulence genes are at the top of the heatmap. Labels are at the tips of maximum parsimony method implemented in kSNP3 (Figure 1). The label colors denote the source of isolates (green for poultry genomes). iTOL was used to build this heat map.

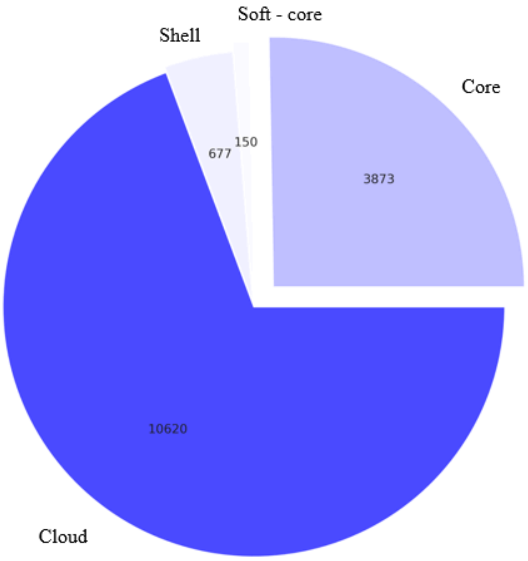

Figure S3. Pie plots of gene content in core, soft core, shell and cloud genomes describing the pan genome for *S*. Minnesota. The core genome is defined as genes present in 25.2% of isolates; soft core, shell and cloud genomes are defined as 0.9%, 4.4% and 69.5% respectively. Number of genomes in pan genome is 107.
